# Wnt/β-catenin and D2 receptor signaling crosstalk in disease mechanisms

**DOI:** 10.1515/biol-2025-1385

**Published:** 2026-10-12

**Authors:** Peikun Zhang, Yuanyan Ge, Xu Zhang

**Affiliations:** College of Basic Medicine, Xinjiang Second Medical College, Karamay, 834000, China

**Keywords:** D2DR, GSK3β, signaling crosstalk, neurological disorders, Wnt

## Abstract

Wnt/β-catenin signaling is a fundamental regulator of development, tissue homeostasis, and disease progression. Recent studies have suggested functional interactions between Wnt signaling and dopamine D2 receptor (D2DR) pathways, with the AKT–GSK3β axis emerging as a key point of convergence that influences β-catenin stability and transcriptional activity. Through this signaling interface, D2DR may shape diverse biological processes, including neuronal survival, synaptic remodeling, and tumor progression. Importantly, the functional consequences of Wnt–D2DR crosstalk appear to be highly context-dependent, differing substantially between neurological disorders and colorectal cancer. In parallel, advances in organoid systems, single-cell technologies, and real-time biosensing approaches are beginning to provide mechanistic insights into the dynamic regulation of these pathways. Although therapeutic strategies directly targeting Wnt–D2DR interactions remain unavailable, growing evidence supports their potential relevance to disease-specific therapeutic interventions. Therefore, a more comprehensive understanding of this signaling network may provide new opportunities for both mechanistic investigation and translational development.

## 1 Introduction

The Wnt/β-catenin signaling pathway has long been recognized as a central regulator of developmental processes and tissue homeostasis [1], 2]. Its activity is tightly controlled through coordinated regulation of β-catenin stability, nuclear translocation, and transcriptional output. Although the core components of this pathway have been extensively characterized, accumulating evidence indicates that its regulation extends beyond canonical ligand–receptor interactions and involves integration with other signaling systems [2].

Recent studies have suggested that G protein-coupled receptor (GPCR) signaling may represent an additional layer of control over Wnt pathway activity [3]. Among these receptors, the dopamine D2 receptor (D2DR) has attracted increasing attention due to its well-established role in neural function and emerging involvement in tumor biology [4], 5]. D2DR signaling may also influence Wnt/β-catenin activity through shared intracellular signaling mediators, indicating potential functional crosstalk between these two pathways. However, current findings remain fragmented, and the molecular basis, disease-specific characteristics, and potential biological significance of Wnt–D2DR crosstalk have not yet been systematically summarized.

One potential point of convergence lies in the AKT–GSK3β signaling axis, which plays a critical role in controlling β-catenin degradation [6]. Modulation of GSK3β activity has been shown to directly influence Wnt signaling output, while D2DR activation can regulate this axis through Gi/o protein–dependent mechanisms [7]. This overlap suggests that D2DR signaling may indirectly shape Wnt pathway dynamics by altering β-catenin stability [6], 8]. Nevertheless, the interaction between Wnt and D2DR signaling is unlikely to be mediated by the AKT–GSK3β axis alone. Emerging evidence suggests that additional intracellular regulators and alternative signaling pathways may also contribute to this crosstalk, although their precise mechanisms and biological significance remain to be fully elucidated.

Beyond intracellular signaling mechanisms, spatial and temporal regulatory processes may further shape the integration of Wnt and D2DR pathways [9]. Variations in receptor localization, signal duration, and feedback regulation are likely to influence whether this interaction promotes or suppresses downstream transcriptional responses [10]. Such context-dependent regulation may differ across tissues and disease states, giving rise to distinct regulatory networks in neurological disorders and colorectal cancer [11]. A better understanding of these dynamic regulatory mechanisms will not only improve our understanding of Wnt–D2DR crosstalk but also provide a conceptual basis for future mechanistic investigations and translational studies.

Although previous reviews have discussed Wnt signaling or D2DR signaling individually, few have provided a comprehensive synthesis of their molecular crosstalk, context-dependent regulation across diseases, translational implications, and emerging experimental approaches within a unified framework. In this review, we integrate current evidence on Wnt/β-catenin–D2DR crosstalk to provide a unified perspective on two signaling systems that have traditionally been studied separately. We summarize the molecular mechanisms underlying this interaction, compare its context-dependent roles in neurological disorders and colorectal cancer, discuss emerging experimental approaches, including organoid models and biosensing technologies, and highlight current knowledge gaps and future research priorities. By integrating evidence across different research fields, this review provides an integrative framework for understanding the role of Wnt–D2DR crosstalk in disease mechanisms and its translational potential.

## 2 Literature search strategy

A structured PubMed search was conducted to identify studies on Wnt/β-catenin–D2DR crosstalk and signaling mechanisms shared by the two pathways, covering publications from database inception to May 31, 2026. Two complementary search strategies were used. The first combined Wnt-related terms (“Wnt,” “Wnt signaling,” “Wnt/β-catenin,” “beta-catenin,” or “β-catenin”) with D2DR-related terms (“dopamine D2 receptor,” “dopamine D2 receptors,” “D2 dopamine receptor,” “D2 dopamine receptors,” “D2 receptor,” “D2 receptors,” “D2DR,” or “DRD2”). The second combined the same D2DR-related terms with shared signaling components (“AKT,” “GSK3beta,” “GSK3 beta,” “GSK3β,” “beta-catenin,” or “β-catenin”). No lower publication-date limit was applied to retain mechanistically important earlier studies.

The two searches yielded 44 and 256 records, respectively (300 records in total). After removal of 35 duplicates, 265 unique records underwent title and abstract screening. Studies addressing direct Wnt–D2DR interactions, D2DR-associated regulation of AKT/GSK3β/β-catenin or related signaling components, or mechanistically relevant disease contexts were retained. After 170 records were excluded for insufficient relevance, 95 publications were retained for further assessment and evidence synthesis. Reference lists of relevant original studies and reviews were also screened to identify additional landmark, methodological, and contextual publications (Figure 1).

**Figure 1:**
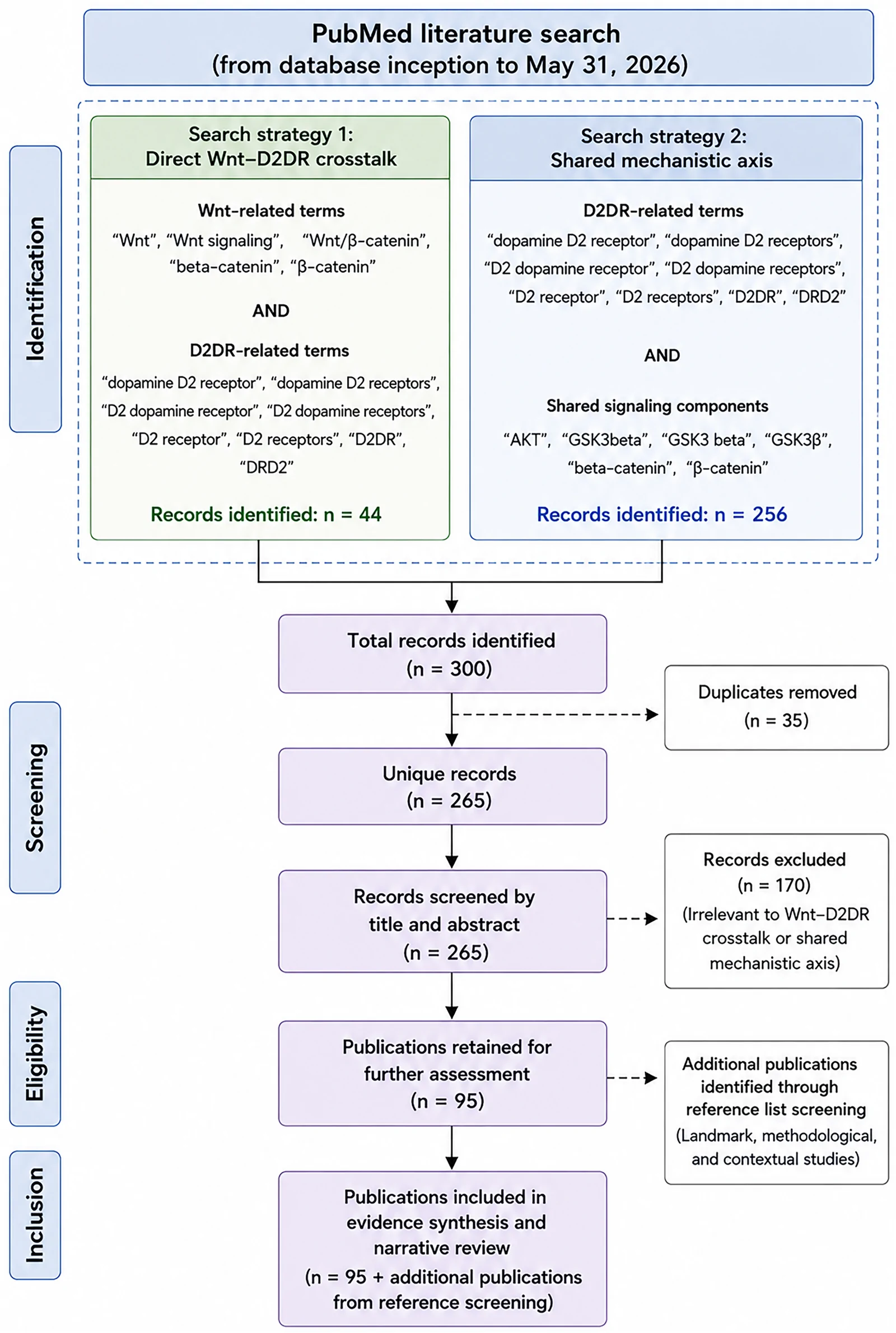
Literature search and study selection strategy.

To minimize selection bias, studies were evaluated according to mechanistic relevance rather than whether their findings supported a predetermined Wnt–D2DR relationship. Direct, indirect, context-dependent, and qualifying evidence was considered where relevant. Original studies were prioritized for mechanistic and disease-specific conclusions, whereas reviews were used primarily for background and citation tracking. Recent evidence was emphasized, while landmark earlier studies were retained when necessary. Evidence based mainly on preclinical models or indirect associations was interpreted cautiously.

## 3 Basis of core signaling pathways

### 3.1 Recent advances in Wnt signaling

Wnt signaling is now widely recognized as a multilayered regulatory system rather than a simple ligand–receptor cascade [2], 7]. Beyond its established roles in development and tissue homeostasis, recent studies have emphasized its disease-specific involvement in cell fate determination and pathological progression, particularly through the β-catenin–dependent branch of the pathway [1], 7]. The core architecture of canonical Wnt signaling involves ligand binding to Frizzled (Fzd) and LRP5/6 receptors, followed by β-catenin stabilization (Figure 2, left).

**Figure 2:**
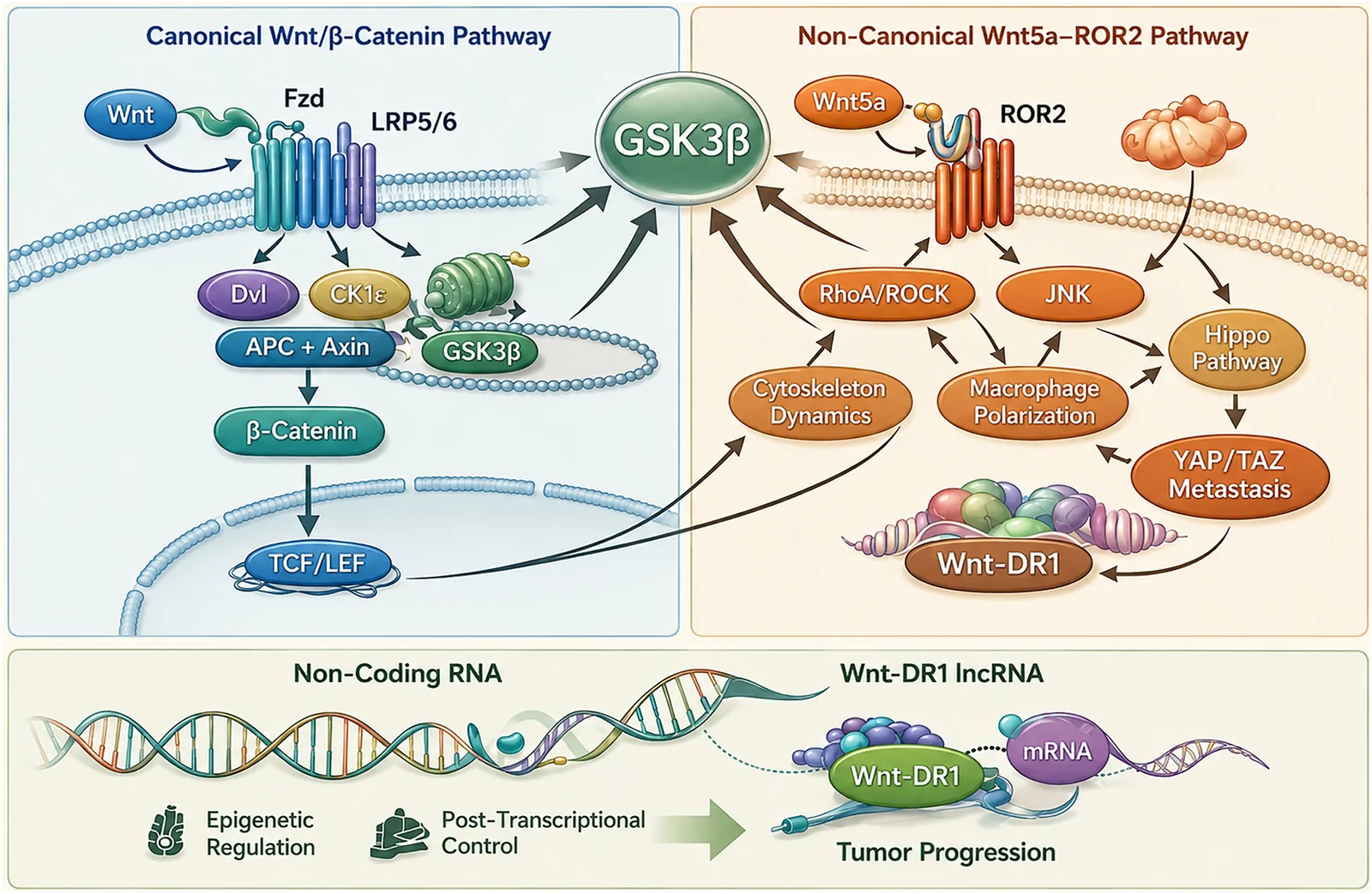
Overview of canonical and non-canonical Wnt signaling pathways, highlighting GSK3β as a central hub and the roles of Wnt5a–ROR2 signaling and non-coding RNAs in regulating downstream pathways and tumor progression.

Within canonical Wnt signaling, increasing attention has been given to the dynamic assembly of intracellular signaling complexes. Dishevelled (Dvl) acts as a central scaffold linking receptor activation to downstream signaling events, while CK1ε-mediated phosphorylation further regulates Dvl activity, subcellular localization, and signal propagation [12], 13]. These regulatory processes ultimately converge on the β-catenin destruction complex (APC–Axin–GSK3β), which controls β-catenin stability and transcriptional output [7]. Dysregulation of this module, particularly through APC mutations, contributes to sustained pathway activation in colorectal cancer [1].

In contrast, non-canonical Wnt signaling exhibits distinct regulatory features. The Wnt5a–ROR2 axis (Figure 2, right) has emerged as a key mediator of cytoskeletal remodeling and cellular plasticity. This pathway can activate downstream effectors such as RhoA–ROCK and JNK, thereby influencing cell migration, inflammatory responses, and metabolic adaptation [10], 14], 15]. In addition, Wnt5a–ROR2 signaling can antagonize canonical Wnt activity in a context-dependent manner and has been shown to interact with the Hippo pathway to regulate YAP/TAZ-mediated transcription and tumor progression [16].

Another emerging layer of Wnt regulation involves non-coding RNAs. Long non-coding RNAs such as Wnt-DR1 can modulate Wnt signaling at multiple levels, including epigenetic regulation, post-transcriptional control, and protein localization (Figure 2, bottom) [17], 18]. Increasing evidence suggests that aberrant expression of Wnt-associated lncRNAs contributes to epithelial–mesenchymal transition (EMT), tumor progression, and metastatic behavior, highlighting their role in shaping the disease-specific regulation of Wnt signaling [19], 20].

### 3.2 Emerging concepts in D2DR signaling

The dopamine D2 receptor (D2DR) is a class A G protein-coupled receptor (GPCR) that regulates dopaminergic signaling through both Gi/o-dependent and β-arrestin–mediated pathways [4]. Upon activation, D2DR inhibits adenylyl cyclase and reduces intracellular cAMP levels, while β-arrestin–dependent signaling engages downstream kinases such as AKT, ERK, and glycogen synthase kinase 3β (GSK3β) (Figure 3) [21]. Notably, the two splice variants, D2S and D2L, exhibit functional divergence, with D2S preferentially mediating G protein signaling and D2L more closely associated with β-arrestin–dependent pathways [4], 22]. Collectively, these signaling branches establish D2DR as a multifunctional signaling regulator that coordinates diverse intracellular kinase networks beyond its classical role in dopaminergic neurotransmission.

**Figure 3:**
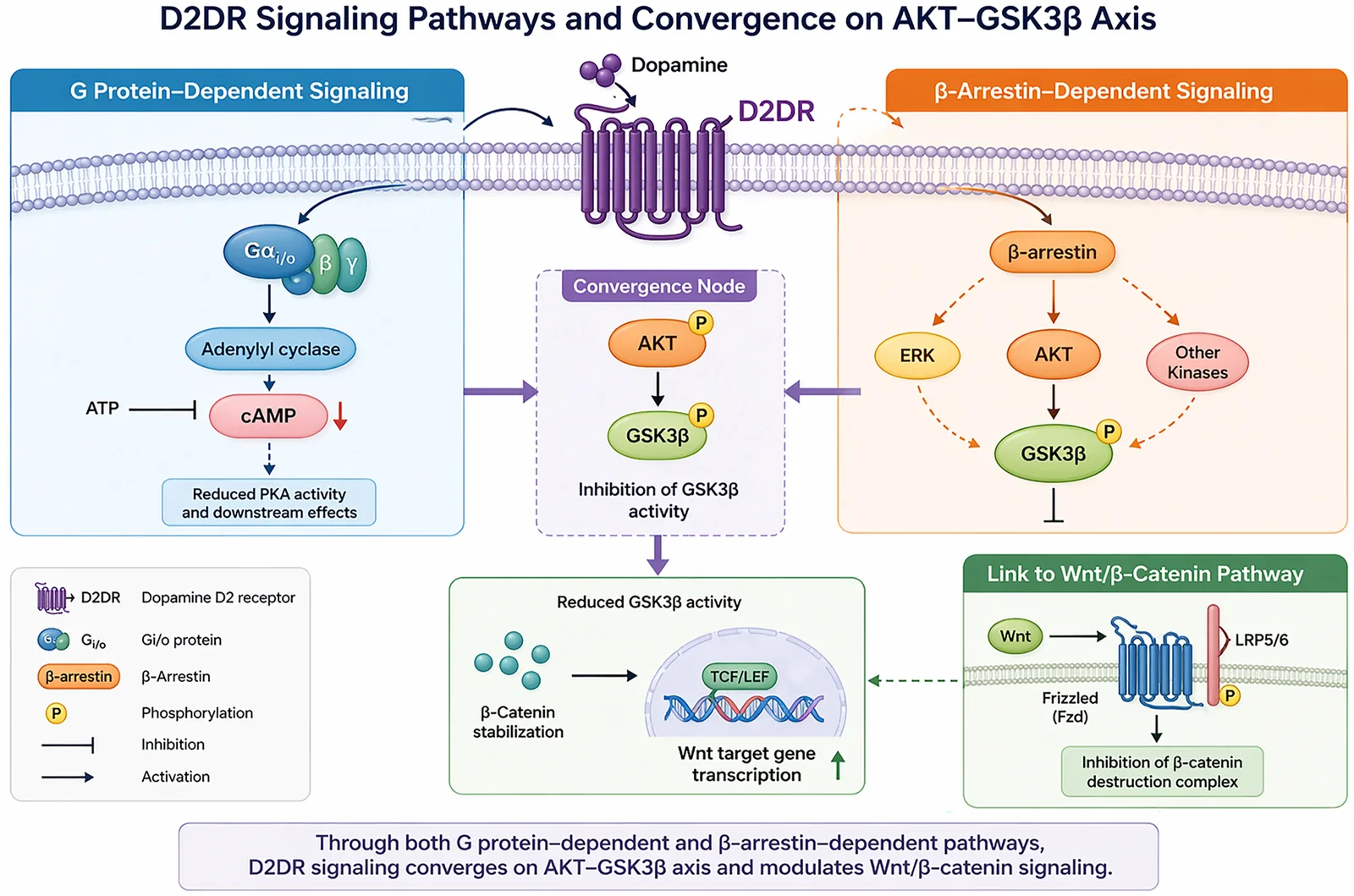
Overview of D2DR signaling pathways, illustrating Gi/o-mediated inhibition of cAMP and β-arrestin–dependent activation of AKT–GSK3β signaling, along with emerging therapeutic strategies targeting these pathways.

Current evidence indicates that the functional relevance of D2DR signaling extends beyond dopaminergic neurotransmission and is closely linked to pathways controlling cell fate and disease progression. In particular, the convergence of D2DR signaling on AKT and GSK3β provides a mechanistic bridge to the Wnt/β-catenin pathway, where GSK3β acts as a central regulatory node controlling β-catenin stability [7]. This overlap suggests that D2DR may influence Wnt signaling dynamics through modulation of β-catenin turnover and transcriptional activity, thereby contributing to processes such as neuronal plasticity and tumor progression. Consequently, D2DR should be viewed as part of a broader signaling network that intersects with Wnt-dependent regulatory systems rather than functioning in isolation.

Beyond its established neurological functions, D2DR signaling has also been implicated in tumor cell survival, proliferation, and disease progression [23]. Through both Gi/o protein–dependent and β-arrestin–mediated signaling, D2DR coordinates multiple intracellular signaling networks, including AKT, ERK, and GSK3β, in a context-dependent manner [24], 25]. This broad signaling capacity suggests that D2DR functions not only as a dopamine receptor but also as an important regulator of diverse cellular signaling processes. Among the signaling pathways functionally associated with D2DR, Wnt/β-catenin signaling has attracted increasing attention and will be discussed in the following section [2], 25].

## 4 Crosstalk mechanisms

### 4.1 Molecular interaction interfaces

The crosstalk between Wnt and D2DR signaling may involve multiple shared molecular interfaces rather than a single regulatory pathway, enabling signals from both pathways to converge on common intracellular regulators. Among these interfaces, glycogen synthase kinase 3β (GSK3β) represents one of the best-characterized convergence nodes and serves as a central regulator of β-catenin stability. In the canonical Wnt pathway, in canonical Wnt signaling, Dishevelled (Dvl) promotes receptor-associated signaling events that disrupt the activity of the β-catenin destruction complex, thereby reducing β-catenin degradation and promoting its stabilization. [18], 26]. In parallel, D2DR activation modulates GSK3β through receptor-associated signaling mechanisms, further influencing β-catenin turnover and transcriptional activity (Figure 4I) [18], 26].

**Figure 4:**
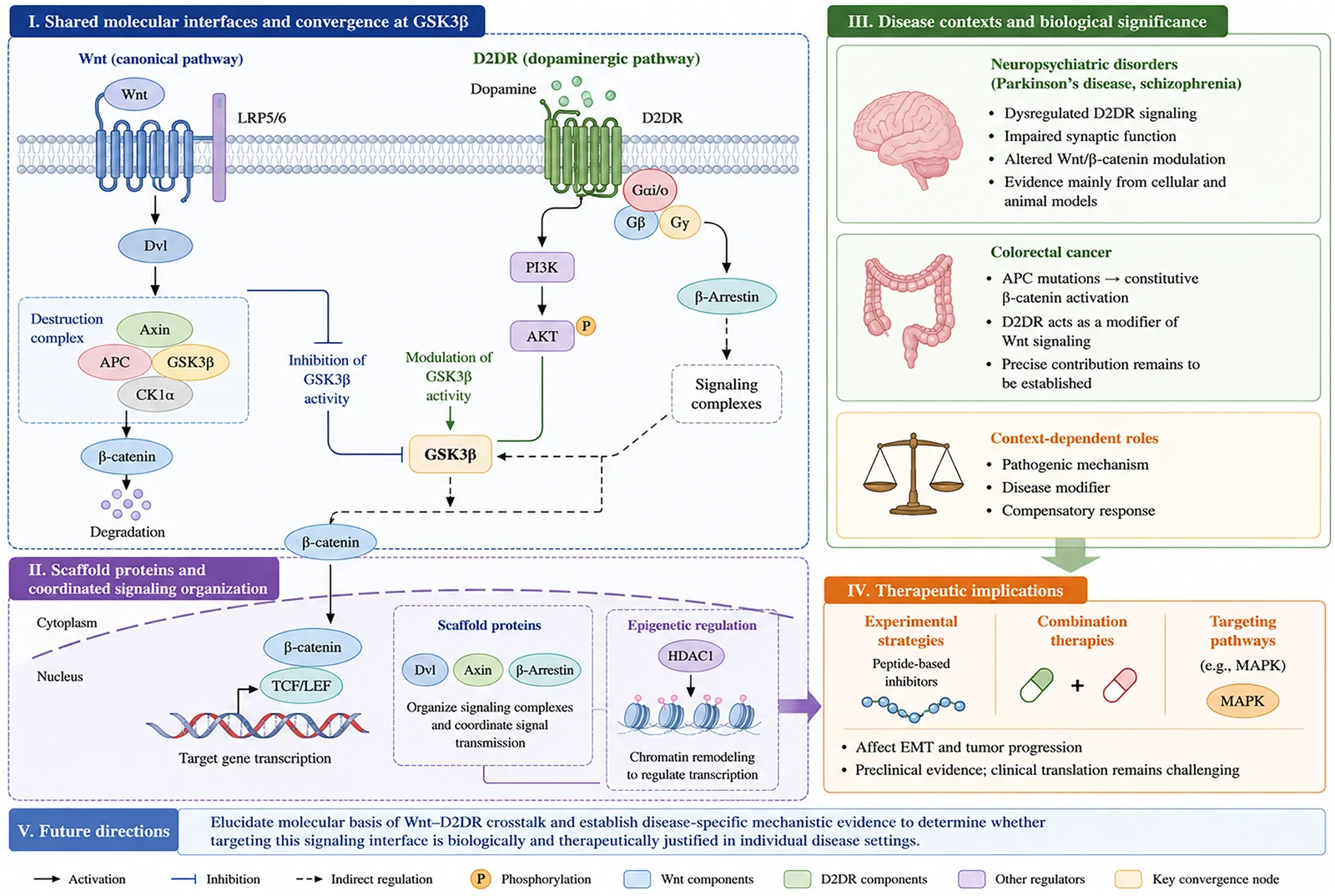
Molecular interaction interfaces underlying Wnt–D2DR crosstalk. GSK3β integrates canonical Wnt and D2DR signaling, while scaffold proteins and HDAC1 coordinate β-catenin–dependent transcription.

Although the AKT–GSK3β axis remains the best-characterized mechanism underlying Wnt–D2DR crosstalk, it is unlikely to fully account for the complexity of signaling integration between these pathways [7]. Additional regulatory components, including scaffold proteins such as Axin and β-arrestin, receptor-associated signaling complexes, and non-canonical Wnt signaling, are also likely to contribute to this process in a context-dependent manner (Figure 4I) [7], 27]. The relative importance of these mechanisms appears to vary across different cell types and disease settings, suggesting that Wnt–D2DR crosstalk is better viewed as a dynamic regulatory network than as a single linear signaling cascade [7]. A more comprehensive understanding of how these regulatory components interact will be important for clarifying the molecular basis of Wnt–D2DR signaling in both physiological and pathological conditions.

Beyond kinase-mediated regulation, Wnt–D2DR crosstalk also depends on the coordinated organization of receptor-associated signaling complexes and scaffold proteins, which spatially integrate signaling events and facilitate efficient signal transmission [7], 28]. β-Arrestin promotes the assembly of signaling complexes downstream of D2DR, whereas Dishevelled (Dvl) and Axin organize core components of the Wnt pathway to regulate β-catenin stability [28], 29]. Although these scaffold proteins function within distinct signaling pathways, they collectively contribute to the organization and propagation of intracellular signals. In addition, epigenetic regulators such as histone deacetylase 1 (HDAC1) further modulate β-catenin-dependent transcription through chromatin remodeling, linking membrane-associated signaling events to downstream transcriptional responses. Together, these regulatory components suggest multiple potential levels of signaling coordination, ranging from receptor-associated complexes to transcriptional regulation (Figure 4II).

Alterations in Wnt–D2DR signaling interfaces have been implicated in the pathogenesis of multiple diseases; however, the underlying mechanisms and biological significance of this crosstalk differ considerably across disease contexts (Figure 4III) [25], 30]. In neuropsychiatric disorders such as Parkinson’s disease and schizophrenia, dysregulated D2DR signaling has been associated with impaired synaptic function and altered modulation of Wnt/β-catenin-related pathways, although current evidence is derived predominantly from cellular and animal models and remains largely correlative rather than mechanistically definitive (Figure 4III, upper) [31], 32]. By contrast, colorectal cancer is primarily driven by genetic alterations in core components of the Wnt pathway, particularly APC mutations that result in constitutive β-catenin activation [11]. Under these conditions, D2DR is more likely to function as a modifier of Wnt signaling than as a primary driver of tumor initiation, although its precise contribution remains to be established (Figure 4III, middle) [23], 25]. These observations indicate that the biological significance of Wnt–D2DR crosstalk is determined not only by the signaling pathways involved but also by the dominant molecular alterations underlying individual diseases [11]. Defining whether this crosstalk represents a pathogenic mechanism, a disease modifier, or a compensatory response will therefore be essential for understanding its biological significance and therapeutic potential (Figure 4III, lower) [33].

Although accumulating evidence suggests that Wnt–D2DR crosstalk may represent a therapeutic target, current therapeutic strategies remain largely exploratory (Figure 4IV). Experimental approaches, including peptide-based inhibitors and combination therapies, have demonstrated encouraging results in preclinical models by modulating interconnected signaling pathways such as Wnt and MAPK, thereby influencing biological processes including epithelial–mesenchymal transition and tumor progression [34], 35]. However, the clinical translation of these findings is limited by the incomplete understanding of the molecular mechanisms governing Wnt–D2DR crosstalk, as well as its marked heterogeneity across different tissues and disease contexts [36]. Future progress will therefore depend not only on further elucidating the molecular basis of Wnt–D2DR signaling but also on establishing disease-specific mechanistic evidence to determine whether targeting this signaling interface is biologically and therapeutically justified in individual disease settings (Figure 4V).

### 4.2 Functional synergies

Temporal regulation allows signaling outputs to vary with the intensity and duration of receptor activation, thereby facilitating cellular adaptation to different physiological and pathological conditions (Figure 5I). Moderate D2DR activation promotes GSK3β inhibition and β-catenin stabilization, leading to enhanced β-catenin–dependent transcriptional responses [37]. In contrast, sustained or excessive receptor stimulation activates feedback mechanisms, including receptor internalization and alternative signaling pathways such as Wnt/Ca^2+^ signaling, which attenuate β-catenin–dependent signaling [37], 38]. Rather than generating fixed signaling outputs, these dynamic transitions may shift the balance between pathway activation and feedback inhibition, resulting in context-dependent functional responses [39].

**Figure 5:**
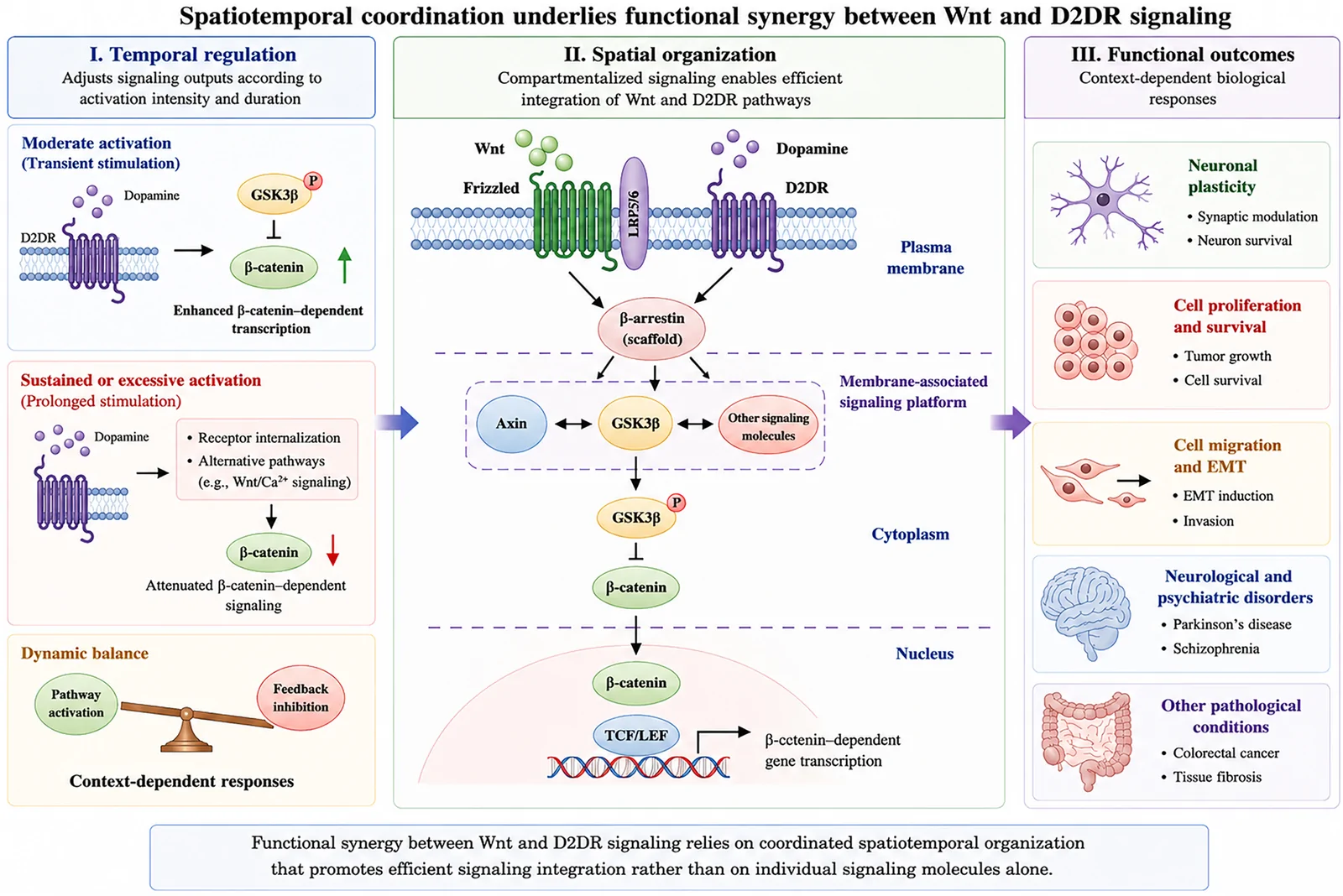
Spatiotemporal coordination of Wnt–D2DR signaling enables efficient signaling integration and context-dependent biological responses.

Spatial organization provides an additional level of functional coordination by restricting signaling events to defined subcellular compartments, thereby enhancing signaling specificity (Figure 5II) [40], 41]. Following D2DR activation, β-arrestin functions as a scaffold protein that promotes the assembly of membrane-associated signaling complexes, enabling receptors and downstream signaling molecules to interact within localized signaling platforms [42]. Rather than directly coupling D2DR to the canonical Wnt pathway, these signaling platforms facilitate coordinated communication among shared signaling components and create favorable conditions for signaling integration [29]. In parallel, canonical Wnt signaling regulates the intracellular localization and activity of GSK3β and β-catenin, allowing signaling information to be transmitted sequentially from the plasma membrane through the cytoplasm before reaching the nucleus to regulate β-catenin-dependent gene transcription [2]. Such compartmentalization not only enhances signaling specificity but may also contribute to distinct biological responses across cellular and pathological contexts [43]. Consequently, coordinated spatiotemporal organization may contribute to functional integration between Wnt and D2DR signaling and help shape their biological effects (Figure 5III).

## 5 Disease-associated mechanisms

### 5.1 Diseases of the nervous system

Accumulating evidence suggests that Wnt–D2DR crosstalk contributes to the regulation of neuronal survival and synaptic function, with emerging evidence implicating this interaction in neural circuit homeostasis. In Parkinson’s disease (PD), impaired Wnt/β-catenin signaling has been associated with reduced dopaminergic neuron survival. Dysregulated D2DR signaling may further influence this pathway through shared intermediates such as GSK3β, potentially affecting β-catenin-dependent transcription involved in neuronal survival and synaptic maintenance (Figure 6A) [31], [44], [45], [46]. Rather than functioning as independent signaling pathways, Wnt and D2DR appear to converge on common regulatory nodes that coordinate neuronal resilience, synaptic integrity, and adaptive responses to neurodegenerative stress (Figure 6A). This convergence may be particularly important in PD, where dopaminergic neurons exhibit exceptional vulnerability to mitochondrial dysfunction, oxidative stress, and impaired proteostasis, thereby increasing their dependence on coordinated pro-survival signaling [47], 48]. However, current evidence supporting this interaction is derived predominantly from cellular and animal models, and direct evidence demonstrating that altered Wnt–D2DR crosstalk is a primary driver of neurodegeneration in patients remains limited [49]. In addition to neuronal survival, disruption of coordinated Wnt and D2DR signaling has been associated with impaired synaptic plasticity and neural circuit stability, although these abnormalities may also reflect broader alterations in disease-associated signaling networks rather than the direct consequence of Wnt–D2DR dysfunction alone.

**Figure 6:**
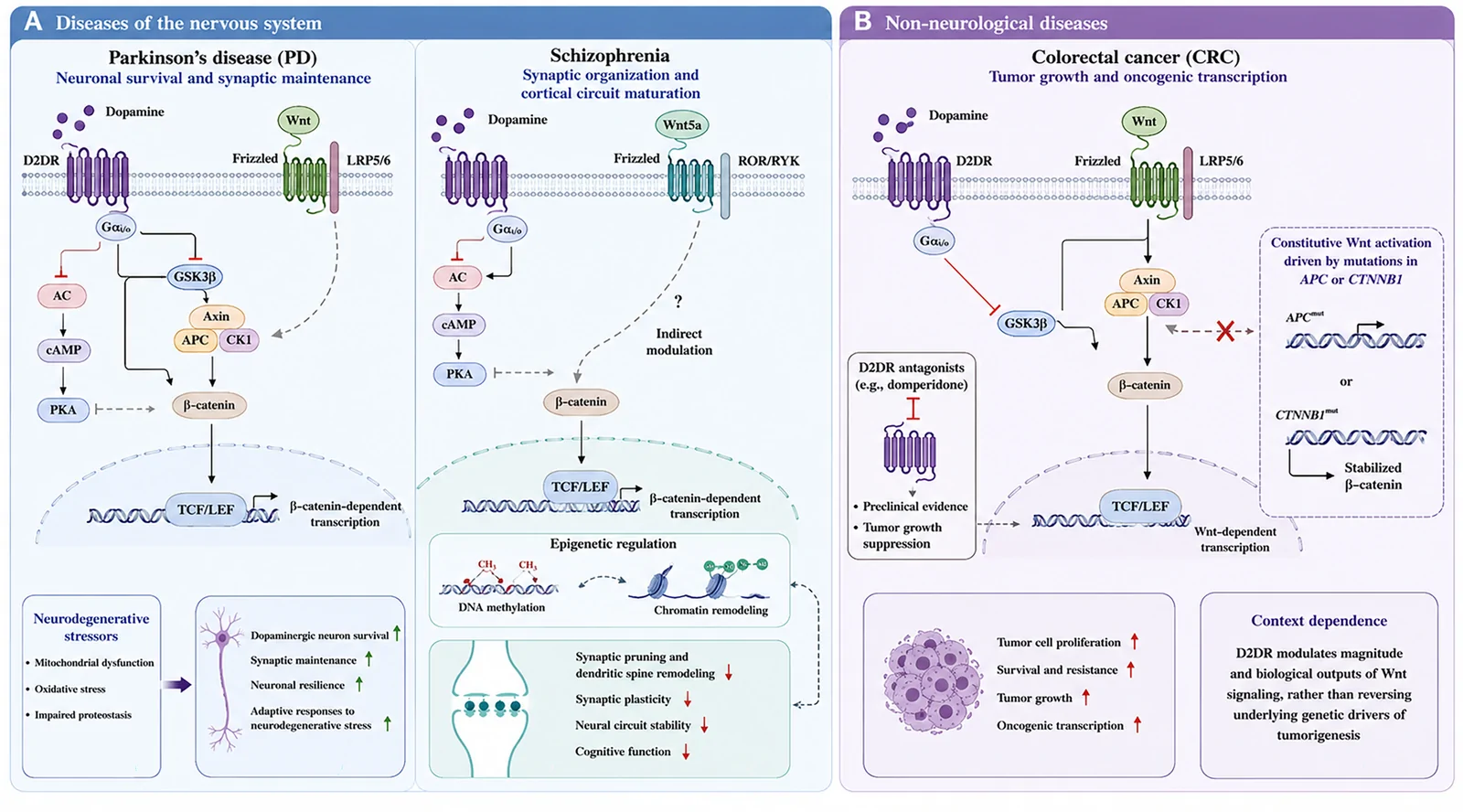
Context-dependent roles of Wnt–D2DR crosstalk in neurological and non-neurological diseases. Wnt–D2DR crosstalk differentially regulates neuronal homeostasis and tumor progression through disease-specific molecular contexts and signaling outputs.

Whereas Wnt- and D2DR-related signaling in Parkinson’s disease has been discussed mainly in the context of neuronal survival, their potential interaction in schizophrenia is more closely associated with synaptic organization and cortical circuit maturation [50], 51]. Dysregulated Wnt5a signaling, together with altered D2DR activity, has been associated with impaired synaptic pruning and dendritic spine remodeling [52], 53]. Although the molecular interaction between these pathways remains incompletely understood, current evidence suggests that excessive D2DR activation may indirectly influence Wnt signaling through the AC–cAMP–PKA pathway, thereby modulating β-catenin activity and Wnt signaling [32], 54]. Epigenetic mechanisms, including DNA methylation and chromatin remodeling, may further regulate both D2DR signaling and Wnt pathway activity, contributing to persistent synaptic dysfunction [32], 55]. These regulatory processes may be particularly important during cortical circuit development, where precise coordination of synaptic remodeling is essential for maintaining normal cognitive function. However, considerable variability exists among experimental models and patient cohorts, making it difficult to determine whether dysregulated Wnt–D2DR crosstalk represents a primary pathogenic mechanism of schizophrenia or an adaptive consequence of neural circuit remodeling during disease progression.

### 5.2 Non-neurological diseases

Colorectal cancer (CRC) provides an excellent model for understanding how D2DR signaling modulates constitutively activated Wnt signaling. Aberrant activation of canonical Wnt signaling, driven predominantly by mutations in *APC* or *CTNNB1*, results in constitutive β-catenin activation and sustained oncogenic transcription (Figure 6B) [11], 56]. Consequently, CRC differs fundamentally from Parkinson’s disease in its Wnt signaling context. Whereas impaired Wnt activity may compromise neuronal survival in Parkinson’s disease, canonical Wnt signaling in CRC is frequently constitutively activated. D2DR may therefore act primarily as a modifier of downstream signaling output rather than as a determinant of pathway activation. Current evidence suggests that D2DR may influence β-catenin activity through shared signaling intermediates, particularly GSK3β, thereby potentially modulating Wnt-dependent transcription and tumor-associated cellular behaviors (Figure 6B) [56], 57]. However, the contribution of D2DR-mediated regulation is likely to depend on the underlying genetic landscape of individual tumors, particularly the mutational status of *APC* and *CTNNB1*, and therefore remains incompletely understood.

Consistent with these observations, preclinical studies have shown that pharmacological inhibition of D2DR suppresses colorectal tumor growth, supporting a functional role for D2DR in CRC. Importantly, whether these antitumor effects are mediated directly through Wnt signaling remains to be fully established [58], 59]. Nevertheless, the therapeutic significance of this interaction should be interpreted cautiously, as current evidence is derived predominantly from cellular and animal models, with limited validation in clinical settings. Accordingly, D2DR-targeted interventions are more likely to modulate downstream signaling output than to reverse the underlying genetic drivers of tumorigenesis (Figure 6B) [60]. Future therapeutic development will therefore require a more comprehensive understanding of D2DR-dependent regulation of Wnt signaling, together with the identification of patient populations and molecular contexts most likely to benefit from D2DR-targeted interventions.

## 6 Therapeutic targeting of Wnt–D2DR crosstalk

Increasing evidence suggests that Wnt–D2DR crosstalk may represent a promising therapeutic target in multiple diseases. However, no pharmacological agents have been specifically developed to directly target this signaling interface. Current therapeutic approaches mainly focus on either D2DR or Wnt signaling, and their effects on Wnt–D2DR crosstalk remain largely indirect and incompletely understood [30], 61]. Therefore, current evidence is derived primarily from studies of D2DR-targeting drugs, Wnt pathway inhibitors, and shared signaling regulators, which collectively provide preliminary support for exploring disease-specific therapeutic strategies.

Current pharmacological evidence relevant to the Wnt–D2DR signaling axis is derived primarily from agents that independently modulate either D2DR or Wnt signaling. Among these, D2DR agonists, including cabergoline and bromocriptine, remain the standard first-line therapy for prolactinomas and hyperprolactinemia by suppressing prolactin secretion and inhibiting tumor growth through D2DR activation (Table 1, row 1, 2) [62], 67]. In parallel, several Wnt-targeted agents, including PORCN inhibitors such as WNT974 (LGK974) and ETC-159, as well as Wnt5a-mimetic agents such as Foxy-5, have entered early-phase clinical trials for Wnt-related malignancies, including colorectal cancer and other solid tumors (Table 1, row 4–6) [68], [69], [70]. Although these agents have demonstrated promising therapeutic activity by targeting their respective pathways, increasing experimental evidence suggests that modulation of one pathway may indirectly influence the activity of the other through shared regulatory mechanisms [30]. Nevertheless, whether these pharmacological effects truly reflect therapeutic modulation of Wnt–D2DR crosstalk remains uncertain and requires further mechanistic investigation.

**Table 1:** Representative pharmacological agents and their potential relevance to Wnt–D2DR signaling crosstalk.

Reference	Agent	Primary target	Disease context	Development stage	Potential relevance to Wnt–D2DR crosstalk
[62]	Cabergoline	D2DR agonist	Prolactinoma, hyperprolactinemia	Approved	Activates D2DR signaling and may indirectly modulate β-catenin activity through the AKT–GSK3β pathway
[63]	Bromocriptine	D2DR agonist	Prolactinoma, hyperprolactinemia	Approved	Alters D2DR-dependent signaling and may influence downstream Wnt-related signaling regulators
[64]	ONC201	DRD2 antagonist	Glioblastoma, solid tumors	Phase II clinical trials	Inhibits DRD2 signaling and may affect β-catenin-dependent transcription through shared signaling intermediates
[65]	WNT974 (LGK974)	PORCN inhibitor	Wnt-dependent solid tumors, CRC	Phase I clinical trials	Suppresses Wnt ligand secretion and may indirectly alter D2DR-associated signaling responses
[66]	ETC-159	PORCN inhibitor	Gastrointestinal and Wnt-driven cancers	Phase I clinical trials	Inhibits canonical Wnt signaling and affects downstream β-catenin activity
NCT03883802 (clinical trial)	Foxy-5	Wnt5a mimetic peptide	Breast cancer, colorectal cancer	Phase I/II clinical trials	Mimics non-canonical Wnt5a signaling and may influence signaling balance associated with D2DR-mediated pathways

Despite encouraging progress in both D2DR- and Wnt-targeted therapies, translation of these pharmacological advances into Wnt–D2DR-directed therapeutic strategies remain at an early stage. Most available evidence has been generated from cellular systems, animal models, and a limited number of organoid-based studies, whereas clinical evidence that modulation of Wnt–D2DR crosstalk improves patient outcomes is lacking [71], 72]. Furthermore, accumulating evidence indicates that the biological consequences of Wnt–D2DR crosstalk are highly context-dependent, varying with disease type, genetic background, and tissue microenvironment. For example, in Parkinson’s disease, therapeutic intervention is expected to restore impaired Wnt/β-catenin signaling to promote dopaminergic neuron survival, whereas in colorectal cancer, constitutive activation of Wnt signaling caused by APC or CTNNB1 mutations requires suppression rather than activation of the same pathway [56], 73]. Consequently, pharmacological modulation of Wnt–D2DR crosstalk is unlikely to produce universal therapeutic benefits across different diseases, highlighting the importance of disease-specific therapeutic strategies and patient stratification. Furthermore, limited tissue specificity, potential systemic toxicity, and unintended off-target effects remain important barriers to clinical translation, with inadequate blood–brain barrier permeability posing an additional challenge in neurological applications.

## 7 Emerging experimental platforms and translational applications

Although progress has been made in characterizing potential interactions between Wnt and D2DR signaling, conventional cell culture systems and animal models have limitations in recapitulating the complex cellular interactions and disease-specific signaling environments of human tissues. Emerging experimental platforms, particularly organoid-based models and co-culture systems, provide more physiologically relevant systems for investigating context-dependent Wnt–D2DR signaling while better preserving tissue architecture and cellular heterogeneity (Table 2, row 1) [74], 75]. Nevertheless, these models cannot fully recapitulate the complexity of the native tissue microenvironment, including immune interactions, vascularization, and long-range neural connectivity, highlighting the need for complementary experimental and clinical validation.

**Table 2:** Emerging experimental strategies for Wnt–D2DR crosstalk.

Reference	Experimental platform	Outstanding question addressed	Mechanistic insight provided	Remaining challenges
[74], 75]	Organoid-based and co-culture models	How can disease-specific Wnt–D2DR crosstalk be faithfully modeled?	Reconstruct disease-specific signaling microenvironments and validate context-dependent Wnt–D2DR crosstalk	Incomplete immune, vascular, and neuronal microenvironment reconstruction
[76], 77]	Live-cell imaging and FRET biosensors	How are Wnt–D2DR signaling dynamics regulated?	Reveal the spatiotemporal dynamics of GSK3β/β-catenin signaling	Limited molecular coverage and technical complexity
[78], 79]	CRISPR perturbation + single-cell transcriptomics	Which molecules function as core regulatory nodes?	Establish causal regulatory relationships and identify functional signaling hubs	High computational complexity and limited validation *in vivo*
[71], 80]	Patient-derived samples and clinical validation	Under which molecular contexts is Wnt–D2DR therapeutically relevant?	Define disease-specific therapeutic windows and biomarkers	Limited prospective clinical validation

Beyond improving disease modeling, emerging experimental technologies provide new opportunities to resolve several key mechanistic questions that remain unanswered in Wnt–D2DR research. For example, live-cell imaging and FRET-based biosensors could help determine whether and how D2DR activation influences GSK3β and β-catenin dynamics, including whether such effects are rapid and reversible or involve sustained pathway remodeling (Table 2, row 2) [76]. CRISPR-mediated perturbation combined with single-cell transcriptomics further enables functional interrogation of candidate regulatory nodes identified in Wnt–D2DR signaling networks, allowing investigators to distinguish core signaling hubs from secondary downstream responses (Table 2, row 3) [78], 81]. These approaches may also clarify how shared signaling components contribute to distinct biological outcomes across disease contexts, thereby enabling direct experimental testing of the context-dependent models of Wnt–D2DR crosstalk proposed above.

Despite the rapid development of these experimental platforms, translation of mechanistic discoveries into clinical applications remains challenging. Most current evidence supporting therapeutic modulation of Wnt–D2DR crosstalk is derived from organoid, cellular, and animal models, whereas direct clinical validation remains limited [72]. Moreover, the disease-specific nature of Wnt–D2DR signaling suggests that therapeutic responses are likely to vary according to disease type, molecular subtype, and cellular microenvironment, making universal therapeutic strategies unlikely to be effective. Future studies should therefore prioritize integrating advanced experimental platforms with patient-derived samples and clinical investigations to identify the molecular contexts in which modulation of Wnt–D2DR crosstalk is biologically and therapeutically meaningful, thereby facilitating the development of more precise and disease-context-specific therapeutic strategies.

## 8 Summary and outlook

The crosstalk between Wnt signaling and dopamine D2 receptor (D2DR) pathways has emerged as an important framework for understanding both neurological disorders and tumor progression [7], 8]. Rather than acting entirely independently, these signaling systems may converge on shared regulatory nodes, particularly GSK3β and AKT, providing potential points of integration between extracellular stimuli and intracellular responses [82], 83]. Increasing evidence indicates that this coordinated signaling influences cellular behavior, disease susceptibility, and therapeutic responses in a context-dependent manner [36]. By integrating current knowledge of molecular mechanisms, disease-specific functions, emerging experimental platforms, and therapeutic strategies, this review highlights Wnt–D2DR crosstalk as a multifaceted signaling network with potential relevance across diverse pathological conditions.

In disease settings, the biological consequences of Wnt–D2DR crosstalk are highly context-dependent. In neurological disorders such as Parkinson’s disease and schizophrenia, alterations in β-catenin signaling and D2DR activity have each been implicated in neuronal survival, synaptic plasticity, and neural circuit homeostasis, although their direct functional interaction remains incompletely defined [84], 85]. By contrast, in colorectal cancer (CRC), where canonical Wnt signaling is frequently constitutively activated by APC or CTNNB1 mutations, D2DR is more likely to function as a modifier of Wnt signaling output rather than a primary driver of pathway activation [56], 58]. These observations suggest that the biological significance of Wnt–D2DR crosstalk is determined not only by shared signaling mechanisms but also by the molecular and cellular context in which these interactions occur.

Despite these advances, several important questions remain unresolved. The distinct roles of D2DR splice variants (D2S and D2L) in regulating Wnt-dependent transcription, the existence of temporally defined signaling windows, and the limited availability of tools capable of establishing causal relationships within Wnt–D2DR signaling networks continue to constrain current understanding [86], 87]. Addressing these challenges will require the integration of advanced experimental approaches, including structural biology, organoid-based disease models, CRISPR-mediated perturbation strategies, spatial transcriptomics, single-cell analysis, and real-time biosensing technologies [88], [89], [90]. The combination of these complementary platforms may facilitate the identification of key regulatory nodes, clarify context-dependent signaling mechanisms, and provide more direct evidence for the biological significance of Wnt–D2DR crosstalk in human disease.

From a translational perspective, the therapeutic relevance of Wnt–D2DR crosstalk remains to be established. Although several D2DR-targeting agents and Wnt pathway modulators have entered clinical development or routine clinical use, no therapeutic strategy has yet been specifically designed to directly target Wnt–D2DR signaling interactions. Furthermore, the context-dependent nature of this crosstalk suggests that therapeutic responses may vary substantially across different diseases and patient populations. Future efforts should focus on identifying disease-specific intervention points, improving target selectivity, and integrating molecular stratification approaches to facilitate the development of precision therapies based on Wnt–D2DR signaling regulation.

In conclusion, available evidence suggests that interactions between Wnt and D2DR signaling may form a context-dependent regulatory framework relevant to disease-associated cellular processes. Continued advances in mechanistic studies, experimental technologies, and translational research are expected to provide deeper insights into how these pathways cooperate across different biological contexts. A more comprehensive understanding of the molecular principles governing Wnt–D2DR signaling interactions may ultimately enable the development of more effective and individualized therapeutic strategies for neurological disorders, colorectal cancer, and other Wnt–D2DR-associated diseases.
